# Evolutionary patterns of DNA base composition and correlation to polymorphisms in DNA repair systems

**DOI:** 10.1093/nar/gkv197

**Published:** 2015-03-12

**Authors:** Xianran Li, Michael J. Scanlon, Jianming Yu

**Affiliations:** 1 Department of Agronomy, Iowa State University, Ames, IA 50011, USA; 2 Plant Biology Section, School of Integrative Plant Science, Cornell University, Ithaca, NY 14853, USA

## Abstract

DNA base composition is a fundamental genome feature. However, the evolutionary pattern of base composition and its potential causes have not been well understood. Here, we report findings from comparative analysis of base composition at the whole-genome level across 2210 species, the polymorphic-site level across eight population comparison sets, and the mutation-site level in 12 mutation-tracking experiments. We first demonstrate that base composition follows the individual-strand base equality rule at the genome, chromosome and polymorphic-site levels. More intriguingly, clear separation of base-composition values calculated across polymorphic sites was consistently observed between basal and derived groups, suggesting common underlying mechanisms. Individuals in the derived groups show an A&T-increase/G&C-decrease pattern compared with the basal groups. Spontaneous and induced mutation experiments indicated these patterns of base composition change can emerge across mutation sites. With base-composition across polymorphic sites as a genome phenotype, genome scans with human 1000 Genomes and HapMap3 data identified a set of significant genomic regions enriched with Gene Ontology terms for DNA repair. For three DNA repair genes (*BRIP1, PMS2P3* and *TTDN*), ENCODE data provided evidence for interaction between genomic regions containing these genes and regions containing the significant SNPs. Our findings provide insights into the mechanisms of genome evolution.

## INTRODUCTION

DNA base composition is a fundamental genome feature that impacts codon usage, DNA methylation, speciation, genome organization and phylogenetic inference (1–5). Genome sequencing makes it possible to examine overall genome patterns as well as potential mechanisms. Significant research progress has been made in three related areas: DNA base composition (6–8), mutation spectra (9–15) and DNA repair systems (16,17). However, systematic analyses to generate a synthesized framework have not been adequately conducted.

Among these three research areas, the first is the individual-strand base composition. The Chargaff's first parity rule (PR1) of nucleotide base composition in double-stranded DNA (i.e. [A] = [T] and [G] = [C]) was an integral pre-requisite to the Watson–Crick's double-helical model (18). The less-known, second parity rule (PR2) was stated to summarize the observation that for each individual strand of a DNA duplex, [A] ≈ [T] and [G] ≈ [C] (19). Although the validity of PR2 was previously demonstrated at the genome level (8) and various theories have been proposed (6,7), no further large-scale studies using a diverse set of species with sequenced whole genomes have been reported. More critically, no studies on individual-strand base composition across single nucleotide polymorphisms (SNPs) have been reported using a population of related individuals. Because these polymorphic sites constitute the dynamic part of the genome and are most abundant, it would be interesting to study whether there is a pattern, which may contribute to our understanding of the underlying mechanisms for individual-strand base equality.

The second related research area is mutation bias. Base mutation was shown to have a bias in the direction of A:T and that newly emerged low-frequency SNP alleles are typically A:T rich (9,10). Evolutionary modeling research demonstrated the relative effect of mutation rate and fixation probability in shaping human base composition (11). For example, substantial biases towards lower overall GC content were discovered from sequence comparison between human and other primates at both paralogous repeat elements and orthologous genes (12,13). However, genetic relationships of human individuals were not examined in earlier studies where AT frequency was examined across human SNPs (14,15). We can ask whether this change in base frequency will emerge if we compare individual-strand base composition across polymorphic sites between populations separated by a bottleneck event.

In the third research area, fine control of the mutation process in living organisms has been revealed by the elucidation of DNA repair mechanisms in model species and the discovery of hundreds of associated genes in humans (16,17). From an evolutionary perspective, an efficient and effective DNA repair system is essential to the maintenance of genome integrity; mutations in DNA repair genes can lead to hypermutated genomes, severe diseases, and cancers. On the other hand, some nucleotide changes must inevitably escape this surveillance system to provide genetic variability to fuel the evolutionary process. However, evolutionary divergence of these DNA repair genes themselves in different human groups has not been extensively studied. One recent study suggested that low fidelity DNA replication by polymerase ζ is partly responsible for the observed multinucleotide mutation in the human population (20). Contrasting genome-wide sequence polymorphisms between populations separated by a bottleneck would allow us to probe potential roles of DNA repair systems in long-term genome evolution.

In this study, we report two base-composition patterns, PR2 and A&T-increase/G&C-decrease, first at the polymorphic-site level across multiple natural populations and second at multiple sites across progenies derived from common starting individuals in mutation-accumulation experiments. Having identified the same patterns in these mutation-accumulation experiments propel us to examine them in detail in human populations where large data are available. We then establish base-composition value across polymorphic sites as a phenotype of an individual so that the underlying genetics can be examined. Several recent studies demonstrated that population-based genome scans can be used to examine genome phenotypes including recombination rate (21–23), genome size (24) and methylation (25). Accordingly, we conduct genome-wide scans to identify genomic regions that likely underlie the observed base-composition variation in humans. With the significant enrichment of DNA repair genes within these identified genomic regions, we further discuss the potential connection among DNA repair system, genetic divergence, mutation bias, and base-composition pattern.

## MATERIALS AND METHODS

### Genomes of species and data sources

**Figure 1.**
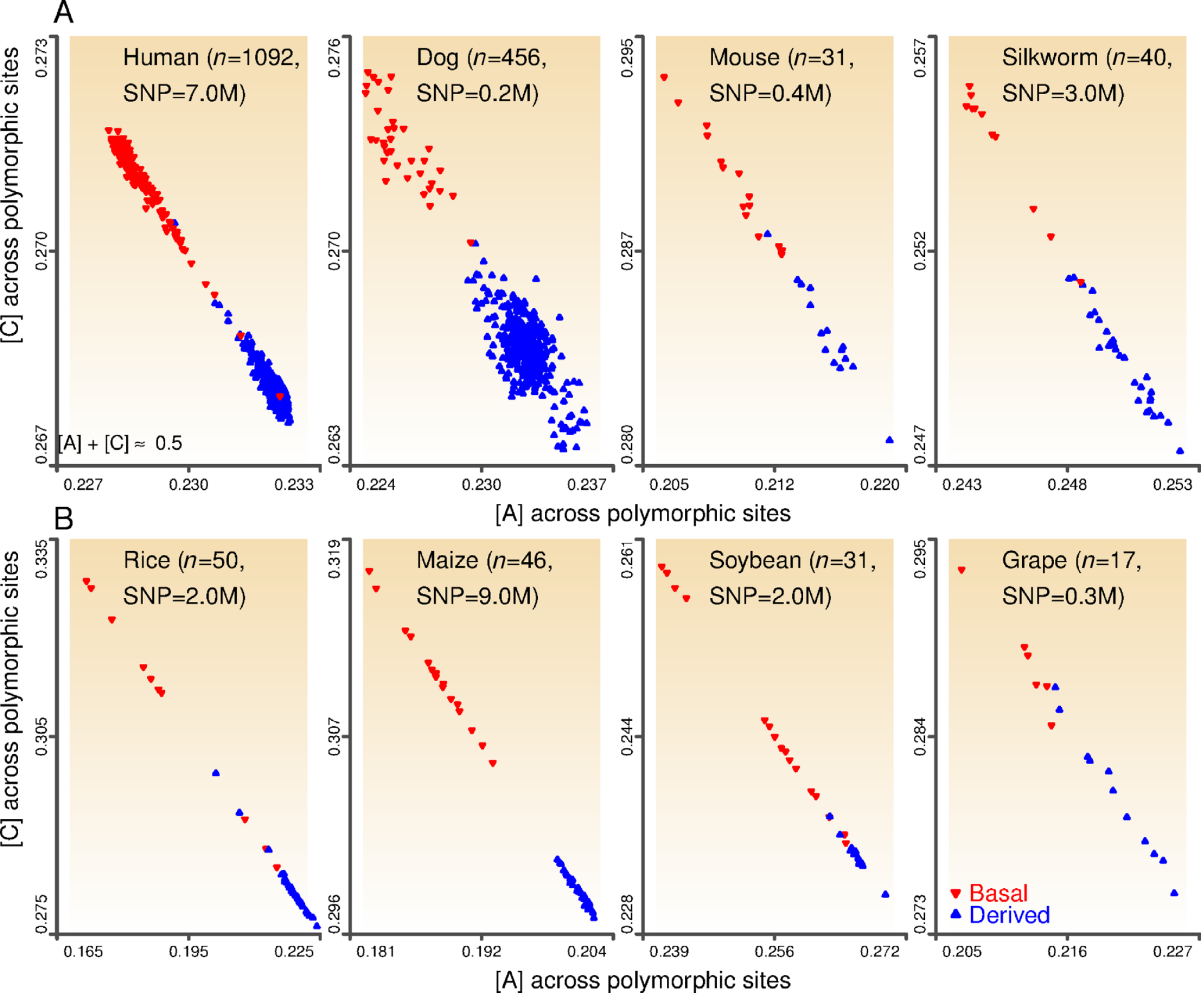
Genome-wide sequence polymorphism patterns in eight comparison sets. Individual-strand A&T-increase and base-composition parity. The A&T-increase pattern is evident by the separation of blue and red triangles, denoting individuals from either basal groups (red, e.g. African-ancestry human samples) or derived groups (blue, e.g. non-African ancestry human samples). Within each plot, individual-strand base composition across polymorphic sites for each individual was plotted as [C] versus [A] to also demonstrate PR2 across polymorphic sites among individuals within a species or two related species ([A] + [C] ≈ 0.5 is due to [A] ≈ [T], [G] ≈ [C], and [A] + [T] + [G] + [C] = 1) (see Supplementary Figure S1 for PR2 at the whole-genome and chromosome level). Relationship among groups was defined in original studies. Sample size (*n*) and SNP numbers are shown.

The genome sequences were obtained from Ensembl Release 66, Ensembl Genomes Release 13, Phytozome v8.0, Wormbase ws230, Vectorbase Release VB-2012–02 or GenBank (Supplementary Figure S1 and Tables S1). For genomes in GenBank, ‘Phase 2’ status was used for selecting the genome to be analyzed, and for other databases, ‘assembled’ status was used. The genome sequence was provided with bases along one strand of DNA. The missing base ‘N’ in the assembled genome was excluded in the analysis. The data for the polymorphic-site analysis were obtained from eight published studies. These data were the only ones with adequate numbers of SNPs genotyped across groups of samples with known evolutionary relationships (Supplementary Table S2). Basal and derived groups were classified according to the original publications. To minimize potential genotyping and imputation biases, only SNPs with more than 80% of genotyped samples were considered, and then a minor allele frequency (MAF) threshold of 5% was applied. The final numbers of SNPs used in our analysis were indicated (Figure 1 and Supplementary Table S2).

### Base composition at the polymorphic-site level

Examples of base-composition calculation across polymorphic sites are provided (Supplementary Figure S2). In actual data with diploid individuals, homozygotes ‘AA’ were counted as 1, heterozygotes as ‘0.5’, and homozygotes of the alternative allele as ‘0’. The sum across all SNPs was recorded as the [A] value for each individual, and the same was done to obtain [T], [C] and [G].

With human 1000 Genomes data (phase 1, version 2), the initial number of SNPs was 38.2M. Trimming data with a MAF threshold of 5% (out of the overall sample) resulted in 7 003 981 SNPs. Hardy–Weinberg equilibrium tests were conducted for each of the 14 origin groups, and only a very small proportion of SNPs (0.04%) showed significant deviation. Plotting of [A] across genome-wide polymorphic sites clearly showed the difference in [A] values between the basal group (ASW, LWK and YRI), and the derived group (CEU, CHB, CHS, CLM, FIN, GBR, IBS JPT, MXL, PUR and TSI) (Supplementary Figure S3). For each autosomal chromosome, we plotted [C] across polymorphic sites against [A] to show the consistent pattern of PR2 and the A&T-increase (Supplementary Figure S4). Principal component analysis (PCA) was carried out with Genome Association and Prediction Integrated Tool (GAPIT) (26).

To verify that findings on base-composition patterns across polymorphic sites are not artifact of the MAF cut-off used, different thresholds were examined for the human 1000 Genomes data, where the sample size is large enough to examine this meaningfully. Consistently, two patterns of base composition across polymorphic sites were evident for all different MAF thresholds: 5% (7.0M SNPs); 1% (11.6M SNPs) and 0.5% (15.4M SNPs) (Supplementary Figures S4 and S5). SNPs with very low MAF, however, may be generated from sequencing error or resulted from somatic mutations in the tissues used for DNA extraction. They are typically screened off for downstream genome-wide association studies (GWAS). As a result, further analyses in our study adopted the 5% MAF threshold after this verification analysis.

### Spontaneous mutation and induced mutation analysis

Three mutation accumulation experiments (over several generations without mutagen applied) and nine induced mutation experiments (with different mutagen-species combinations) were examined for PR2 and A&T-increase patterns (Supplementary Table S3). These experiments were monitored and had known starting materials. Findings from these mutation experiments helped unravel the molecular mechanisms underlying the population- and evolution-level findings of PR2 and A&T-increase patterns.

### Genome-wide association scans for base composition in humans

For human 1000 Genomes data, [A] across 7 003 981 SNPs was used as the genome phenotype for GWAS. Gender was not significant with [A]. In the first genome-wide scan, a mixed model with both fixed covariates (PC2–PC6) and a random kinship component was used (27,28). Parameters in the mixed model were determined by model selection process (27,28). The divergence between the African-ancestry and non-African-ancestry groups was modeled by the kinship matrix that has a block diagonal structure when sorted accordingly. Manhattan plot and quantile–quantile plot (Supplementary Figure S6) were generated with GAPIT. For the mixed model, which is computationally demanding for simulation, the significance threshold *P*-value was determined by Bonferroni correction to be 7 × 10^−9^ (i.e. −log_10_(*P*-value) = 8.1).

In the second genome-wide scan, a linear regression model relating genotypes at each SNP to values of [A] across the population was used to obtain the *F*-test statistics. Another regression model using five covariates (PC2–PC6, because of the near-perfect correlation between [A] and PC1) to control for genetic relationships yielded similar results. The genome-wide threshold value for the linear model was determined with simulations (Supplementary Figure S7). Because large *F*-values were obtained due to large sample size and the strong divergence between African-ancestry and non-African-ancestry samples, it was not feasible to obtain the *P*-values based on distribution. Genomic control by adjusting the inflation factors did not yield a discerning threshold. In this case, we started with a SNP that has a perfect fixation of the opposite alleles in African-ancestry and non-African-ancestry groups. Random switching of the alleles in an increasing proportion of the samples was then used to derive a baseline *F*-test statistics (*F* = 1590) to interpret the overall results. We conducted 1000 simulations at each proportion.

To demonstrate the consistency and validity of these genome-wide association signals, we followed the same procedure to examine the base-composition variation with the HapMap 3 data and conduct a genome-wide scan. A total of 1 457 897 SNPs across 1192 individuals were analyzed. The threshold value of *F*-test statistics (*F* = 1900) was determined by 1000 simulations to interpret the overall results (Supplementary Figure S8). Results of the mixed model analysis of the HapMap 3 were not presented because of the much smaller number of SNPs and because the within population divergence is more sensitive to specific samples than the overall African and non-African divergence.

### Enrichment test of Gene Ontology terms and DNA repair genes nearby GWAS signals

Gene annotation (version 7) was obtained from GENCODE (http://www.gencodegenes.org/). Gene Ontology (GO) annotations (GOC Validation Date: 14 September 2012) of human protein-coding genes were obtained from www.geneontology.org.

We conducted the enrichment test with a series of window sizes centered by significantly associated SNPs as previously described (29). For each window size, the proportion of each GO term associated with genes within the window was compared with its genome-wide proportion. The results from all window sizes are presented in Supplementary Table S4, and from 1.5 Mb in Table 1, which in general showed the enrichment of genes with DNA repair related GO terms.

**Table 1.** Six GO terms are significantly overrepresented within the window^a^ of the identified association signals

GO terms	Odds ratio	*P*-value	Ontology	Annotation (*DNA repair related)
0006281	2.06	4.1 × 10^−7^	Biological process	DNA repair (*)
0008236	2.91	2.9 × 10^−5^	Molecular function	Serine-type peptidase activity
0003684	2.98	3.6 × 10^−5^	Molecular function	Damaged DNA binding (*)
0004252	2.00	3.1 × 10^−4^	Molecular function	Serine-type endopeptidase activity
0006284	3.00	5.3 × 10^−4^	Biological process	Base-excision repair (*)
0006289	2.41	6.9 × 10^−4^	Biological process	Nucleotide-excision repair (*)

^a^Results from the window size of 1.5 Mb are presented here with the order of enrichment significance (*P*-value) and results from other window sizes are presented in Supplementary Table S4.

A total of 170 Human DNA repair genes and its assigned pathways were obtained from http://sciencepark.mdanderson.org/labs/wood/dna_repair_genes.html. The proportion of these genes within each SNP window was compared with the genome-wide proportion. The gene was counted when it was tagged by at least two significantly associated SNPs, and the DNA repair gene was counted when it was tagged by at least three significantly associated SNPs (Supplementary Figure S9). In both tests, we asked whether the proportion of known DNA repair genes within the genomic region is significantly higher than that across the whole genome. The 1.5 Mb window size showed the most significant enrichment, same as the GO term enrichment results.

### Chromatin interaction between regions harboring trait-associated SNPs (TASs) and implicated DNA repair genes

Interaction profiles of genome regions captured by RNA polymerase II of Chromatin Interaction Analysis Paired-End Tags (ChIA-PET) were obtained from the ENCODE project website. We identified the interaction between genomic regions harboring TASs and implicated DNA repair genes for *BRIP1, PMS2P3*, and *TTDN1*. It is likely that more interactions may be discovered with additional ChIA-PET experiments and cell lines.

## RESULTS

### Whole-genome level individual-strand base equality

We first examined the whole-genome level base composition across 2210 sequenced genomes (Supplementary Table S1). The universal conformity of base composition to the individual-strand parity rule (PR2) was consistently observed for different taxonomic groups (Supplementary Figure S1). Because each chromosome contains an individual DNA molecule for species with multiple chromosomes, we further verified PR2 at the chromosome level with 1098 assembled chromosomes (Supplementary Figure S1). An immediate inference from PR2 is that fairly accurate estimates of number and proportion for three other nucleotides can be obtained from one nucleotide of an individual DNA strand. This is true for estimates at both chromosome and whole-genome levels as long as the species contains double-stranded DNA as the hereditary information carrier, which is the case in almost all living organisms except some viruses. Notice that this inference differs from what can be obtained from PR1.

### Genome-wide sequence polymorphism patterns in natural populations

Next, we asked *whether the PR2 conformity holds across polymorphic sites at the population level*. This has not been examined in the past, but patterns in this dynamic and changing part of the genome could provide insight into the mechanisms contributing to the overall base-composition change. To address this question, we analyzed eight datasets (30–37) (Supplementary Table S2), each comprising a large number of SNPs genotyped across a set of individuals. These SNPs are polymorphic sites across the whole set of samples in each original study, and the base-composition value (e.g. [A]) for each individual was calculated as the fraction of SNP alleles that are A (Supplementary Figure S2). The validity of PR2 at the polymorphic-site level is evident in all data sets with the nearly equal values of [A] and [T], and [C] and [G] for an individual. Collectively across individuals, this parity relationship can be shown by the coupling of increasing [A] values with decreasing [C] values, i.e. [A] + [C] ≈ 0.5 (Figure 1 and Supplementary Figure S1).

Another striking finding, however, was that across all populations, base composition separates basal individuals (ancestral source) from those of derived (bottlenecked) groups. Base-composition values calculated across polymorphic sites showed a general A&T-increase/G&C-decrease pattern for individuals in the derived groups, compared with individuals in the corresponding basal (ancestral) groups (Figure 1 and Supplementary Table S2). Here, our use of basal group and derived group is to differentiate them within each comparison according to known species relationship. We acknowledge that both groups have approximately equal evolutionary divergence time from the common ancestor. These comparisons were either within single species (human, dog, mouse, and maize) or between pairs of closely related species (silkworm, rice, soybean, grape and their corresponding progenitor species). For example, human samples more distant from the evolutionary origin (i.e. non-African ancestry) have more base A and T at SNP sites than samples with African ancestry; modern maize accessions have higher A&T content than teosinte accessions; and cultivated rice (*Oryza sativa*) accessions have higher A&T content than accessions from the progenitor species, *O. rufipogon* and *O. nivara*. Correspondingly, samples from the ancestral groups have significantly more base C and G at SNP sites than the derived groups. These comparisons involved species with a range of base-composition values at the genome level, [A] from 0.267 to 0.327, indicating that these findings at the polymorphic-site level are not dependent on base composition at the genome level (Supplementary Figure S1).

To further confirm that these two individual-strand base-composition patterns across polymorphic sites do not depend on the allele frequency threshold for SNPs to be included in calculation, we conducted additional analyses with the human data, for which the sample size is large enough to examine additional minor allele frequency cut-offs (1 and 0.5%). Consistent findings from these analyses supported both base-composition patterns across genome-wide SNPs (Supplementary Figure S4), and across chromosome-wide SNPs (Supplementary Figure S5).

### Genome-wide base composition changes in experimental populations

**Figure 2.**
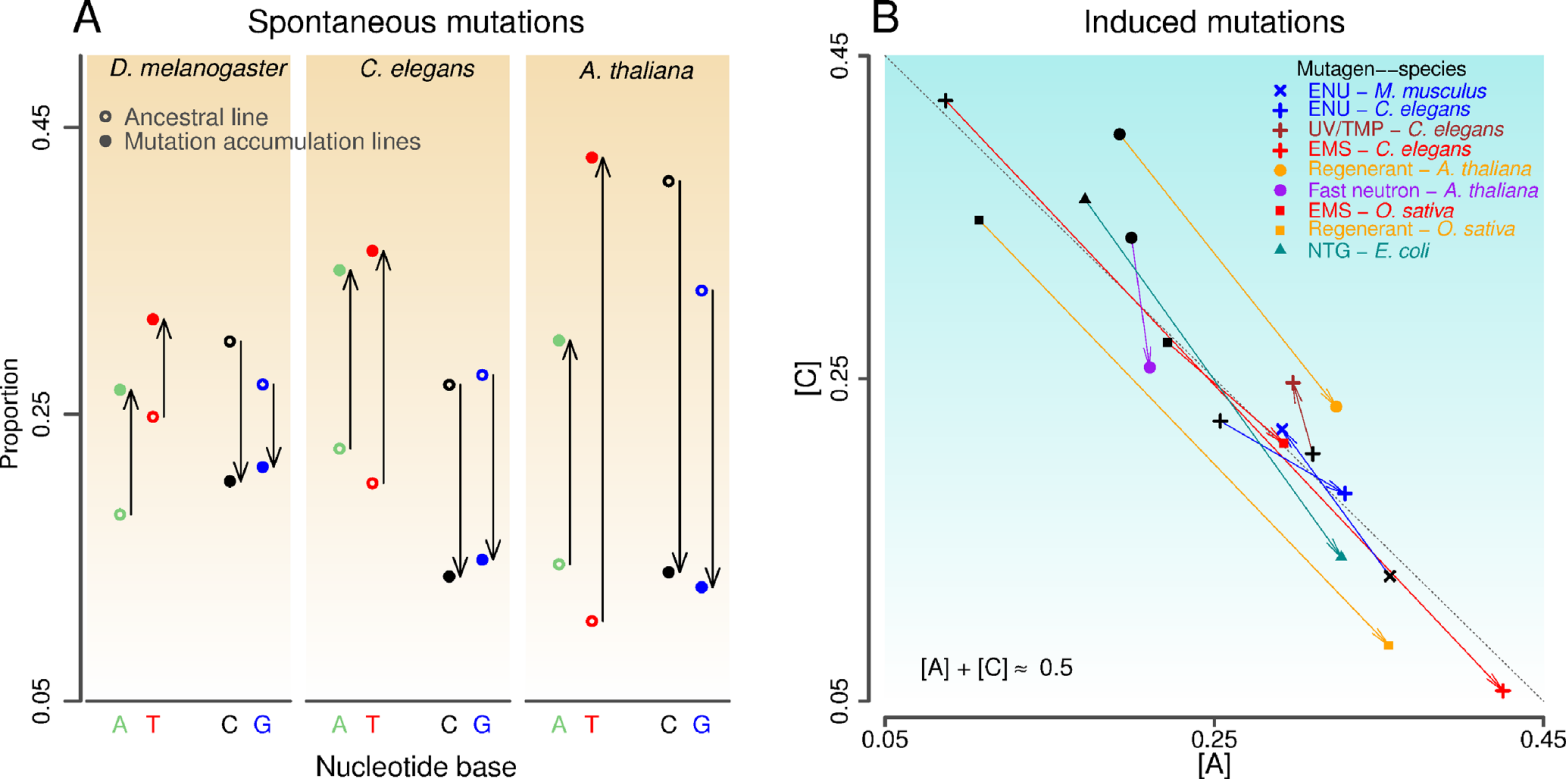
Genome-wide base composition changes across 12 mutation-tracking experiments. (**A**) Individual-strand analysis of three spontaneous mutation experiments to show the base composition change across mutation sites. The derived mutant accumulation lines have higher A&T values than the ancestor lines. Values are similar between [A] and [T] (or between [C] and [G]) for the ancestral line, the same for accumulation lines. (**B**) Individual-strand analysis of 9 different mutagen-species experiments. Base composition, [C] versus [A], follows the diagonal line, indicating the conformity to PR2. A general A&T-increase is also evident in seven experiments. A thin arrow connects the wild-type line (black dot) before mutagen application and the corresponding mutants (colored dot).

The above findings from natural populations reflect base-composition dynamics driven by evolutionary forces, but beg the question: *Will similar base-composition patterns emerge from monitored or intentional mutagenesis experiments*? To this end, we analyzed a series of independent, experimental data of spontaneous and induced mutations (38–46) (mutagenetix.utsouthwestern.edu) (Supplementary Table S3). Analyses conducted in the original studies focused primarily on the mutation spectra (transitions and transversions) derived from both DNA strands. From individual-strand analysis of the sets of spontaneous mutation accumulation lines and the original lines, we found that base composition across mutation sites adheres to PR2 and A&T-increase/G&C-decrease in three model species (Figure 2A). Similarly, our analysis of nine experiments with different mutagen-species combinations also showed a general agreement with the PR2 and A&T-increase/G&C-decrease patterns (Figure 2B). Because different mutagens used in individual experiments involve subsets of the overall mutation/repair systems in a given organism, these collective findings emphasize a common trend and the essential role of mutation and DNA repair mechanisms on base-composition evolution and divergence.

### Base composition across polymorphic sites as genome phenotype

The consistent patterns of PR2 and A&T-increase across polymorphic sites encouraged further investigation of base composition and associated mechanisms. We examined large-scale data from the human 1000 Genomes project (30) in detail. Notice that because of PR2, the proportion of the three other nucleotides can be calculated from any ‘one’ nucleotide of an individual DNA strand. Thus, for the simplicity, the following analyses focused on [A] and the results are essentially the same when [C], [T], [G], [A+T] or [G+C] is analyzed.

First, we found that base-composition values correlate almost perfectly (*r* = −0.98, *P*-value is essentially 0) with the first principal component (PC1) values from the principal component analysis of the SNP data (Supplementary Figure S10), agreeing with the known structure of these samples (Supplementary Figure S3). Because [A] across polymorphic sites has a direct biological interpretation due to its calculation, this near-perfect correlation may be interpreted as follows: the overall separation in base composition across polymorphic sites between African-ancestry and non-African-ancestry individuals (as shown in Figure 1A) can be either revealed directly by the [A] values, or captured by the PC1 from principal component analysis of the same original SNP data (Supplementary Figure S10).

Next, we verified that separation in base composition across polymorphic sites is consistent at the individual chromosome level (Supplementary Figure S4), agreeing with previous reports that similar divergence between human and chimpanzee was observed for each chromosome (47). This verified consistency further suggested that the causal factor is unlikely to be random. The [A] value of an individual, an aggregated measurement calculated across millions of polymorphic nucleotide sites, is very precise. While individual [A] values are calculated across SNP sites arithmetically, these [A] values only approximate the unknown true values. To clarify this point, we conducted a set of subsampling analyses to further demonstrate that different levels of variance component ratios can be estimated when varied numbers of polymorphic sites are used for the [A] calculation (Supplementary Figure S11). These findings supported the notion that base composition calculated across polymorphic sites can be regarded as a genome phenotype, much like height, HDL cholesterol or genome size, whose underlying genetics can be examined by genome wide association studies (48).

### GWAS of base composition in human 1000 Genomes

**Figure 3.**
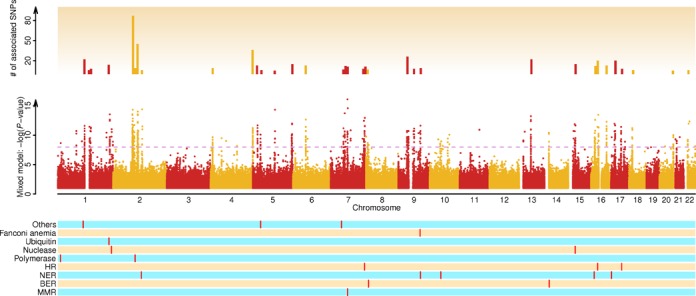
First genome-wide scan with a mixed model to identify genomic regions underlying variation in base composition. The upper panel displays the genomic regions (1 Mb) with five or more TASs detected by the mixed model using genome-wide [A] as trait values of 1092 humans. The middle pane shows the association signals detected by the mixed model between the genome-wide [A] values across polymorphic sites and individual SNPs. The dashed line indicates the threshold determined by Bonferroni correction to control for false positives. The lower panel shows the position and pathway information of known genes (red bars) from different DNA repair mechanisms that are flagged by TASs. MMR, mismatch excision repair; NER, nucleotide excision repair; BER, base excision repair; and HR, homologous recombination.

Although complex evolutionary history is challenging to study and multiple factors are likely to be involved, it should not deter us from making attempts to identify potential genomic regions that drive the divergence in base composition in humans. With this in mind, we first conducted a genome-wide scan with a mixed model controlling for both population structure (fixed effect) and relatedness (random effect) following established methods (27,28) (Figure 3). In this analysis, individual SNPs were tested for association with the genome-wide [A] values while controlling the population structure and relatedness. Given the strong divergence of base composition and the control for population structure, this set of identified genomic regions is more likely to underlie within-population variation in base composition.

Next, we conducted a second genome-wide scan with a linear regression model (Supplementary Figure S12). This scan complements the findings of our first scan because this model does not include population structure so that the major divergence in base composition between humans of African and non-African ancestry can be detected. Because of the unprecedented SNP density of the human 1000 Genomes, this genome scan allowed us to do a top-to-bottom signal screening. In addition to large sample size, focusing on genomic regions with a string of signals reduces the chance of detecting false positives signals. Simulations were performed to establish the threshold to declare significant association signals (Supplementary Figure S7). Our analysis showed that many of the genomic regions with the most significant association signals coincided with regions of elevated allele frequency differences between African-ancestry and non-African-ancestry groups (Supplementary Figure S13).

### Enrichment of certain classes of genes around GWAS signals

The genomic regions flanked by the GWAS signals were further tabulated to answer the following question: *Are any particular groups of annotated genes significantly enriched in these identified genomic regions?* After identifying all GO terms for genes near the GWAS signals, the 1.5Mb window was the most representative size (Supplementary Table S4). Six groups of genes are significantly overrepresented, with *P*-values from 6.9 × 10^−4^ to 4.1 × 10^−7^. Their GO annotations are DNA repair, serine-type peptidase activity, damaged DNA binding, serine-type endopeptidase activity, base-excision repair and nucleotide-excision repair (Table 1).

### DNA repair genes from different pathways and other GWAS signals

The overrepresentation of certain groups of genes found through GWAS scans made it logical to pay close attention to the set of known DNA repair genes in humans (16,17). With the list of 170 known DNA repair genes (Supplementary Table S5), the enrichment test to see whether genomic regions tagged by GWAS signal is more likely to harbor any of these known DNA repair genes is, indeed, significant (Supplementary Figure S9).

In the first genome scan, we identified 21 known genes that are located in the genomic regions flagged by these GWAS signals, or trait-associated SNPs (TASs). These genes are from several DNA repair pathways: base excision repair (BER, e.g. *NEIL2, PARP2* and *APEX1*), mismatch excision repair (MMR, e.g. *PMS2P3*), nucleotide excision repair (NER, e.g. *ERCC3, XPA, ERCC6, ERCC4* and *RPA1*), homologous recombination (HR, e.g. *XRCC2, GIYD1, GIYD2* and *EME1*), DNA polymerases, editing and processing nucleases, ubiquitination and modification and Fanconi anemia (Figure 3 and Supplementary Table S5). Because of the adequate sample size (*n*_1_ = 846 versus *n*_2_ = 246 for African-ancestry samples), non-African ancestry samples were also analyzed independently, and the results were corroborative (Supplementary Figure S14).

Upon further examination of our second genome scan results, a series of 36 known DNA repair genes were tagged by TASs (Supplementary Table S5). The following DNA repair pathways were implicated: BER (e.g. *APLF, MBD4, NEIL3* and *NEIL2*), NER (e.g. *RPA2, UVSSA, XPA, GTF2H1, XPE* and *DDB1*), HR (e.g. *RAD50, NBN, XRCC3, RAD51* and *DMC1*), nonhomologous end-joining (NHEJ), modulation of nucleotide pools, DNA polymerases, editing and processing nucleases, ubiquitination and modification, genes defective in diseases associated with sensitivity to DNA damaging agents, and Fanconi anemia (Supplementary Figure S8).

Genome-wide association studies have provided a wealth of new insights into complex biological phenomena (48). Our research represents the effort to identify genetic factors underlying the base-composition variation at the population level. In three DNA repair pathways (BER, NER and HR), genes flagged by TASs from the linear model were generally located upstream of their particular pathways from genes flagged by TASs from the mixed model, indicating that genes with upstream functions are more likely targets of major divergence (Supplementary Table S5). With two genome-wide scans, we identified not only genomic regions harboring known DNA repair genes but also a series of other genomic regions (Supplementary Table S6). Research continues, so the list of currently known genes is likely incomplete. Our results provide a new resource: genomic regions that may corroborate future findings from other approaches.

### HapMap 3 data and genomic regions tagged by shared TASs

**Figure 4.**
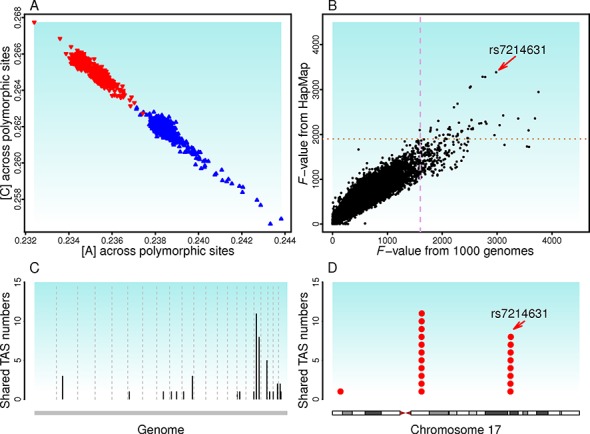
Validation of base-composition divergence and genome scan results with human HapMap 3 data. (**A**) Patterns of PR2 and A&T-increase across samples from the human HapMap 3 are the same as the human 1000 Genomes. (**B**) Consistent GWAS signal strength across common SNPs from the two data sets (*r* = 0.94). Each dot denotes a shared SNP. The dashed lines indicate the thresholds suggested by simulation. The SNP, rs7214631, is among top TASs in both analyses. (**C**) Genomic regions tagged by common TASs with the 1000 Genomes and the HapMap 3 data. (**D**) Human chromosome 17 contains two genomic regions (17q11.2 and 17q23.2) within which multiple common TASs were found. The intensity of black bar shows gene density.

To further corroborate our findings with the human 1000 Genomes data and to show that our approach generalizes to other data, we analyzed the human HapMap 3 data (49). The current HapMap 3 data contain more individuals than the human 1000 Genomes data, but 44% (613) of the individuals in the HapMap 3 were also examined in the human 1000 Genomes. The total number of SNPs in HapMap 3 is about one-fifth of that in 1000 Genomes. As expected, two base-composition patterns recur in HapMap 3: PR2 and A&T difference (Figure 4A).

Next, we conducted a genome scan to identify the major base-composition divergence between African-ancestry and non-African ancestry samples with a linear model. Because of SNP density differences in HapMap 3 and 1000 Genomes, we focused on the GWAS signal strength of the SNPs included in both data sets. Of all 244 TASs identified from the shared SNPs within the HapMap 3 data, 242 were also among the 1470 TASs identified within the 1000 Genomes data. In addition, the relative GWAS signal strength was consistent, which validates the process of mapping base composition as a quantitative trait (Figure 4B and C). Two of these regions in chromosome 17 were particularly interesting: 17q11.2 with 11 shared TASs and 17q23.2 with eight shared TASs (Figure 4D). The SNP rs7214631 was among top TASs detected with both HapMap 3 and 1000 Genomes data; its biological significance is reported in the next section.

### Examples of DNA repair genes associated with base-composition variation

**Figure 5.**
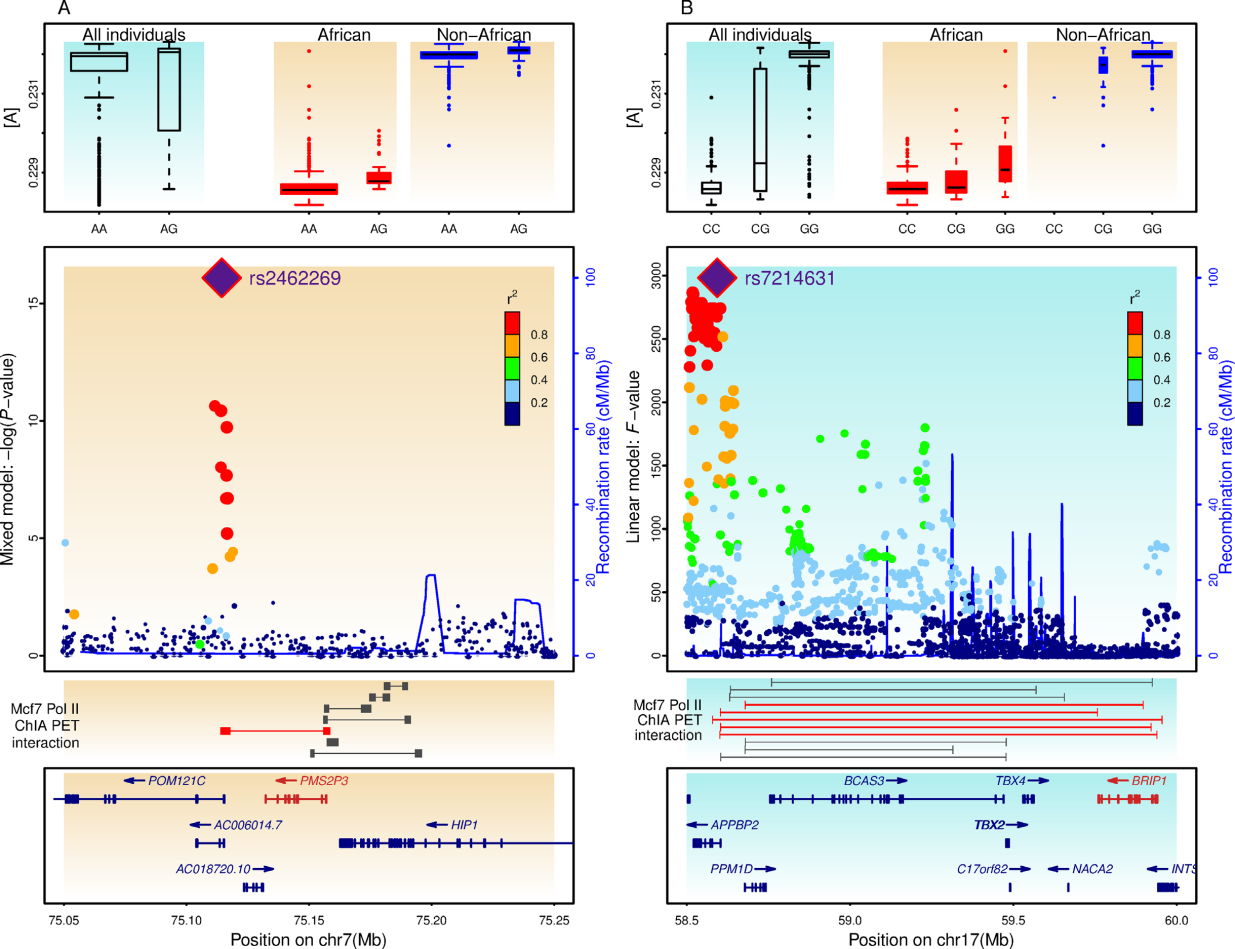
DNA repair genes implicated by trait-associated SNPs (TASs) and potential regulatory interaction. (**A**) *PMS2P3*, encoding a *MutL* homolog, is involved in mismatch excision repair (MMR) pathway. *PMS2P3* is tagged by a TAS (rs2462269) on chromosome 7, underlying the within population variation of base composition as detected by the mixed model. ChIA-PET data indicate that the region harboring rs2462269 and other TASs have long-range chromatin interaction (indicated by connectors) with *PMS2P3*. (**B**) *BRIP1*, encoding a DNA-dependent ATPase and 5′ to 3′ DNA helicase, is a gene in the homologous recombination (NER) pathway. *BRIP1* is tagged by a TAS (rs7214631) on chromosome 17, underlying the divergence of base composition between African-ancestry and non-African-ancestry samples as detected by the linear model. Only the results from 1000 Genomes are shown, although this rs7214631 is also among top TASs detected in HapMap 3 analysis. ChIA-PET data indicate that the region harboring rs7214631 and other TASs have long-range chromatin interaction with *BRIP1*.

*PMS2P3* (postmeiotic segregation increased 2 pseudogene 3) on chromosome 7 (7q11.23) was flagged by a TAS (SNP rs2462269) and a set of other SNPs. *PMS2P3* is among the identified genes of the MMR pathway (16,17) (Figure 5A). No individual had ‘GG’ genotype at this TAS, and heterozyotes ‘AG’ have higher [A] than homozygotes ‘AA’ in both African-ancestry and non-African-ancestry groups. *PMS2P3* encodes a MutL homolog, and the MMR pathway repairs errors in DNA replication and heterologies generated during recombination. Although the exact function of PMS2P3 remains unclear, another MutL homolog (PMS2) is a component of the post-replicative DNA MMR system. PMS2 and MLH form a heterodimer, MutL-α, to resolve the heteroduplex. *PMS2P3* may function through its expression interaction with *PMS2*. The potential interaction between the genomic region harboring association signals and the downstream gene *PMS2P3* was supported by the ChIA-PET tags from the ENCODE project (50) (Figure 5A).

*BRIP1* (BRCA1 interacting protein C-terminal helicase 1) on chromosome 17 (17q23.2), flagged by a TAS (SNP rs7214631), is a DNA-dependent ATPase and 5′ to 3′ DNA helicase required to maintain chromosomal stability (51) (Figure 5B). A large number of nearby SNPs had supporting signals. Although genotype ‘CC’ at rs7214631 is common among individuals with African ancestry, the vast majority of individuals with non-African ancestry have the genotype ‘GG’. *BRIP1* is involved in the repair of DNA double-strand breaks in the homologous recombination pathway. Nonsense and null mutations in *BRIP1* have been implicated in various cancers (52,53). The genomic region harboring these association signals also interact directly with *BRIP1* in RNA Pol II complex, as indicated by ChIA-PET (50), which suggests a potential function in long-rang regulation of the expression of *BRIP1* (Figure 5B).

## DISCUSSION

Our findings at the polymorphic-site level support the no-strand-bias mutation hypothesis proposed to explain PR2 (6,7). If the original mutated nucleotides occur randomly on one DNA strand, base complementation (PR1) during DNA replication would generate a complementary base (e.g. A for T, or T for A) on the newly synthesized strand. This paired emergence would lead to the PR2 when base composition is tallied for each individual strand across mutated sites. While base complementation is highly precise ([A] = [T] and [G] = [C]), the random process of mutation occurring on either strand is not precise, but approaches the expected value of 50%. Subsequently, PR2 across polymorphic sites is approximate ([A] ≈ [T] and [G] ≈ [C]), rather than exact. This explains why the length of the DNA fragment examined needs to be adequately long for PR2 to emerge (7). In this study, we used A&T-increase/G&C-decrease to avoid the confusion with the GC content concept. Although, these concepts are related, GC content is typically used at the species level and for the sum of two bases with the double-strand context (54).

A bottleneck, either human migration out of Africa (55–57) or domestication process (58–60), presumably occurred in these derived groups. Because of the known relationship of the samples, our analysis across SNPs with different frequency spectra indicates that base composition of the derived group moves in the direction of high A&T and low G&C. One way to interpret these findings is that, as a result of lower effective population size, populations after a bottleneck may have fixed A&T mutations more frequently than the basal groups. In other words, given the general AT-bias mutation, the net difference between derived groups and basal groups is a clear pattern of higher A&T in derived groups. An alternative interpretation, however, is that DNA repair genes are likely to affect the number of *de novo* mutations differently in different lineages and a greater total number of mutations could also lead to an increased A&T. It is possible that the base-composition value across polymorphic sites is a secondary link to DNA repair genes that have different efficiency in basal and derived populations (20). Given the equal age of the basal and derived groups in each comparison, our analyses can only reveal the aggregated evolutionary outcome since the divergence of the two groups, and we described this base composition difference by comparing values in derived groups against basal groups. The precise mechanistic explanation for the discovered base-composition patterns across polymorphic sties is challenging to obtain. However, our analysis of the mutation accumulation experiments (38–46) (mutagenetix.utsouthwestern.edu) suggests that both base-composition patterns can emerge under either spontaneous or induced mutations. While base-composition patterns discovered from mutation tracking experiments are vertical comparisons and discovered from derived-versus-basal groups are horizontal comparisons, connection to mutation is the common thread. Nevertheless, detailed population genetic modeling is required to determine whether neutral demographic models with AT-bias mutation can generate the observed patterns between ancestral and bottlenecked populations, or whether other evolutionary forces are needed. Although this modeling research is beyond the scope of the present work, our findings suggest that looking into the dynamic part of the genome across a large number individuals should be considered in addition to the whole-genome base content. Mutation accumulation experiments with starting materials of contrasting DNA repair efficiency are desirable to further demonstrate the connection between mutation and base composition change.

Progress in sequencing technology has produced a huge amount of genome sequences, and the growth of publicly accessible genomic data has not only accelerated progress in pursuing how specific DNA polymorphisms contribute to important morphologic and developmental variations, it also opened new territory to address fundamental questions about the genome itself (61,62). Indeed, several pioneering studies have started unraveling genetic mechanisms underlying different genome features, including recombination rate (21–23), genome size (24) and methylation (25). As with base composition across polymorphic sites, these genome phenotypes are outcomes of a long evolutionary process. From standpoints of evolution and genetics, certain levels of isolation are required between groups of individuals to maintain the relationship between any extant individual's genotype at genomic regions tagged by GWAS and the phenotype (genome features), and over time this isolation enhances the association so that this relationship can be identified. This type of evolution history is generally agreed for the populations in previous studies (21–25) and in the current study.

One feature of the TASs is that although we could identify a known DNA repair gene for many of the tagged genomic regions, these TASs are not within the genes. Targeting multiple haplotypes and rare alleles (63,64) might generate association signals closer to the implicated genes. Although further research may enable this, we probably should not expect such direct TAS hits. Given the critical role of these DNA repair genes, even a minor change in gene expression, let alone function disruption, would affect the efficiency of the DNA repair systems and thus fitness of affected individuals.

Several long-term experiments in *Escherichia coli* have shown that hypermutable phenotypes were generated from mutations within DNA repair genes (65). For example, mutations within the *mutT* gene in *E. coli* lead to much higher A:T->C:G transversion, and thus a significant change in base composition across genome-wide mutation sites (65). Similarly, function disruption in many of these genes could lead to genome instability and various diseases in humans (16,17). On the other hand, modification in pre-existing DNA repair genes was also shown in *E. coli* as an adaptation mechanism (66). At the individual gene level, subtle differences in DNA repair efficiency can be due to multiple layers of regulation, which would leave marks in nearby regions of the DNA repair genes. For example, chromatin remodeling is involved in regulation of transcription, chromosome segregation, DNA replication and DNA repair (67). Indeed, ENCODE projects revealed that long-range regulatory elements can function through chromatin interaction (50), and our analysis identified the interactions between genomic regions harboring TASs and implicated genes (*BRIP1, PMS2P3* and *TTDN1*). Future chromatin interaction experiments with different cell lines at different time points may uncover more such interactions. Sequencing cell lines that derived from starting cells carrying mutations at these DNA repair genes may also generate direct evidence.

In summary, the consistent pattern across eight species comparisons that have available large-scale data highlights the value of examining base-composition dynamics at the population level. These data sets were the only available large-scale data with embedded population divergence during our research, and it is highly desirable to repeat our analysis when new data become available. Base composition difference at the dynamic part of the genome suggests that mutational bias may play an important role in shaping the overall pattern of base-composition variation. By focusing on the outcome of a complicated evolutionary process, our genome scans in human generated relevant information for further research in base-composition change, mutation, and evolution. Through integrating research findings in different fields (1–13,16,17) of genome organization, genome analysis, mutation spectrum, and DNA repair, our analysis of base composition changes and genome-wide scans of base-composition variation established a new approach to examine DNA base-composition evolution and causal biological processes.

## SUPPLEMENTARY DATA

Supplementary Data are available at NAR Online.

SUPPLEMENTARY DATA
