# Supplementary material for: Evolutionary patterns of DNA base composition and correlation to polymorphisms in DNA repair systems
Source: Nucleic Acids Res. 2015 Mar 12;43(7):3614–25. doi: 10.1093/nar/gkv197 (PMC4402523; doi:10.1093/nar/gkv197)
Supplement: SUPPLEMENTARY DATA [file supp_43_7_3614__index.html]

Evolutionary patterns of DNA base composition and correlation to polymorphisms in DNA repair systems — SUPPLEMENTARY DATA 

# Evolutionary patterns of DNA base composition and correlation to polymorphisms in DNA repair systems

## SUPPLEMENTARY DATA

**Files in this Data Supplement:**

- SUPPLEMENTARY DATA
- SUPPLEMENTARY DATA
- SUPPLEMENTARY DATA
- SUPPLEMENTARY DATA
- SUPPLEMENTARY DATA
