# Supplementary material for: Evolutionary patterns of DNA base composition and correlation to polymorphisms in DNA repair systems
Source: Nucleic Acids Res. 2015 Mar 12;43(7):3614–25. doi: 10.1093/nar/gkv197 (PMC4402523; doi:10.1093/nar/gkv197)
Supplement: SUPPLEMENTARY DATA [file supp_gkv197_SupplementaryFigure1-14Tables2-3_03052015.pdf]

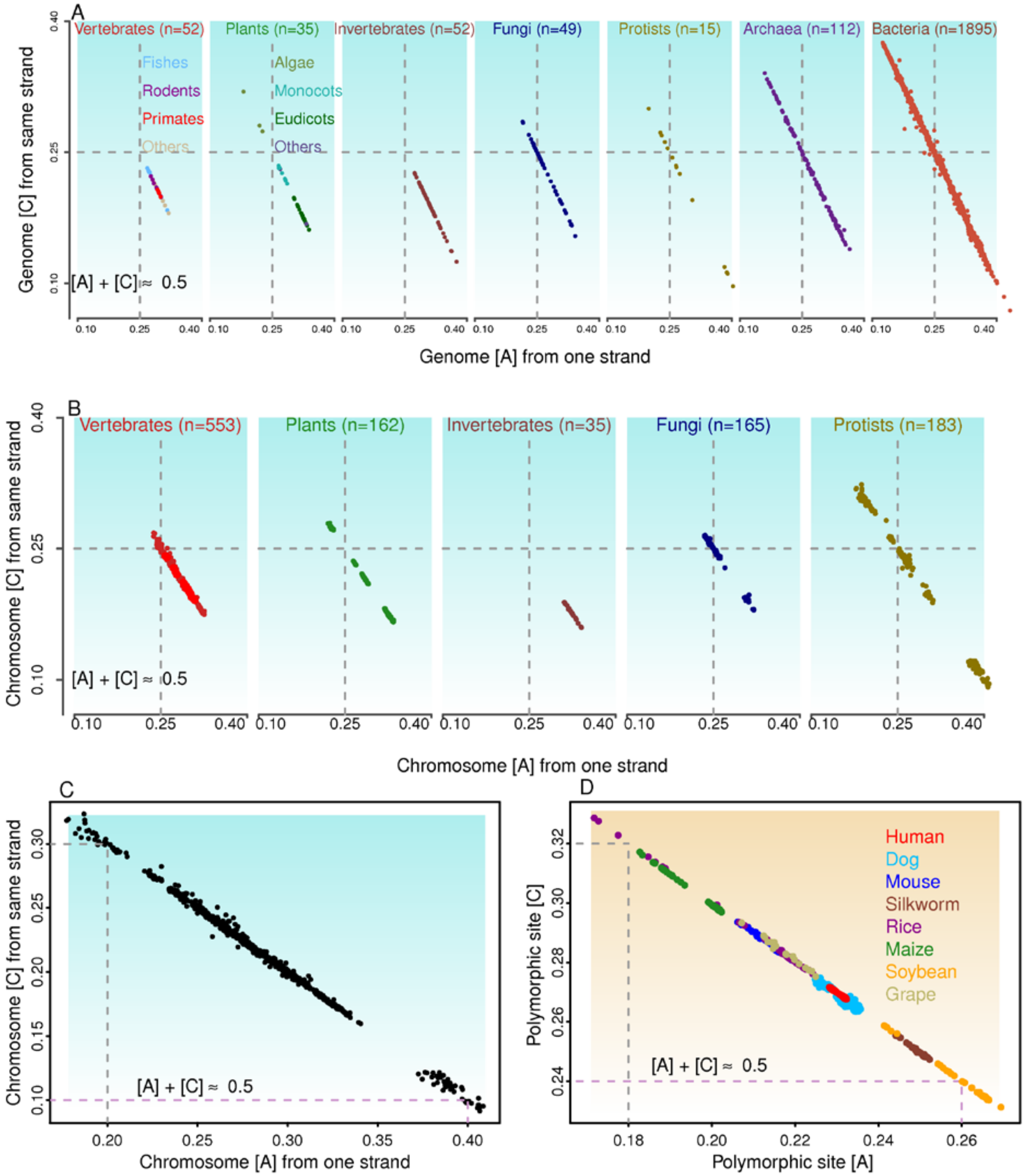

Figure S1. Base composition follows PR2 at the genome, chromosome, and polymorphic-site level. (**A**) Genome-level base composition, [A] and [C], from the same DNA strand across eukaryotic species in different kingdoms. (**B**) Chromosome-level base composition. The number of chromosomes analyzed is shown in parentheses. This analysis was done in species for which sequence assembly is completed at the chromosome level. Within the vertebrate panel, 22 human autosomes and 2 human sex chromosomes are included. (**C**) A combined plot of base composition at the chromosome level across 5 eukaryotic kingdoms. (**D**) A combined plot of base composition across polymorphic sites across 8 species comparison sets.

Sample	Group	SNP genotype matrix	[A]
1	Basal	**A** G **A** C T C T C G C C G G G G C	2/16 = 0.13
2	Basal	**A** G **A** C T C T **A** G C C G G G G C	3/16 = 0.19
3	Basal	C G **A** C T C T **A** G C C G **A** G G C	3/16 = 0.19
4	Derived	**A** G **A** C T **A** T **A** G **A** C T G T G C	5/16 = 0.31
5	Derived	**A** G T T C **A** C **A** G **A** **A** T G T G **A**	6/16 = 0.38
6	Derived	**A** **A** **A** T C **A** T **A** T **A** **A** G G T T C	7/16 = 0.44

Figure S2. Calculation of base composition across polymorphic sites. In this example, 16 SNPs were detected across 6 samples. For simplicity, homozygosity is assumed for all SNP sites and for all samples. The [A] for 3 samples from the derived group is higher than the derived group. The actual number of SNPs in the current study varied from 283,574 in dog to 9,230,417 in maize. The actual number of samples (*e.g.*, an individual human or a grape strain for which a set of SNPs were available for analysis) varied from 17 in grape to 1,092 in human.

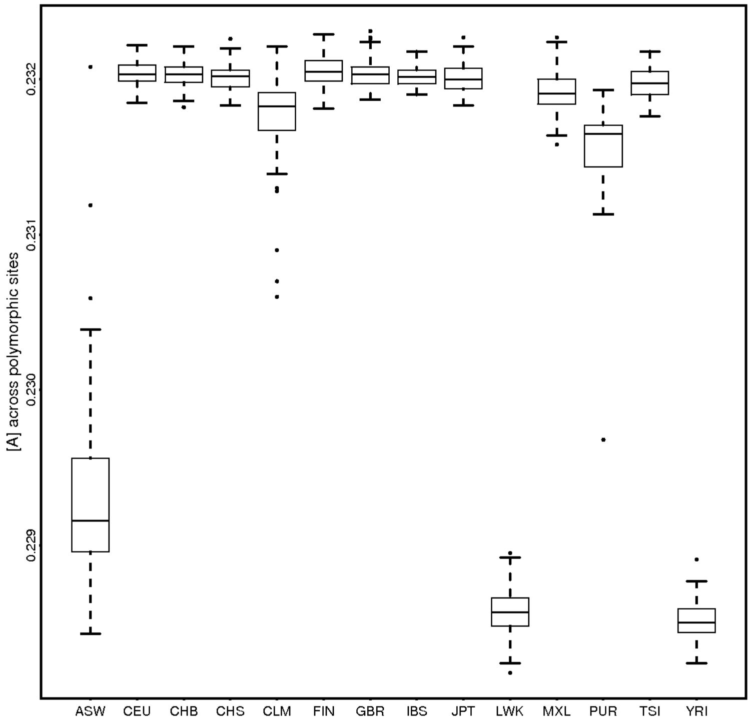

Figure S3. Base composition across polymorphic sites separates human samples. The separation of [A] between the basal group and derived group is evident and shows minimal overlap. The basal (ancestral source) group includes ASW, LWK, and YRI. The derived (bottlenecked) group includes CEU, CHB, CHS, CLM, FIN, GBR, IBS, JPT, MXL, PUR, and TSI. For each sample in the human 1000 Genomes data, base composition [A] was calculated across 7,003,981 SNPs.

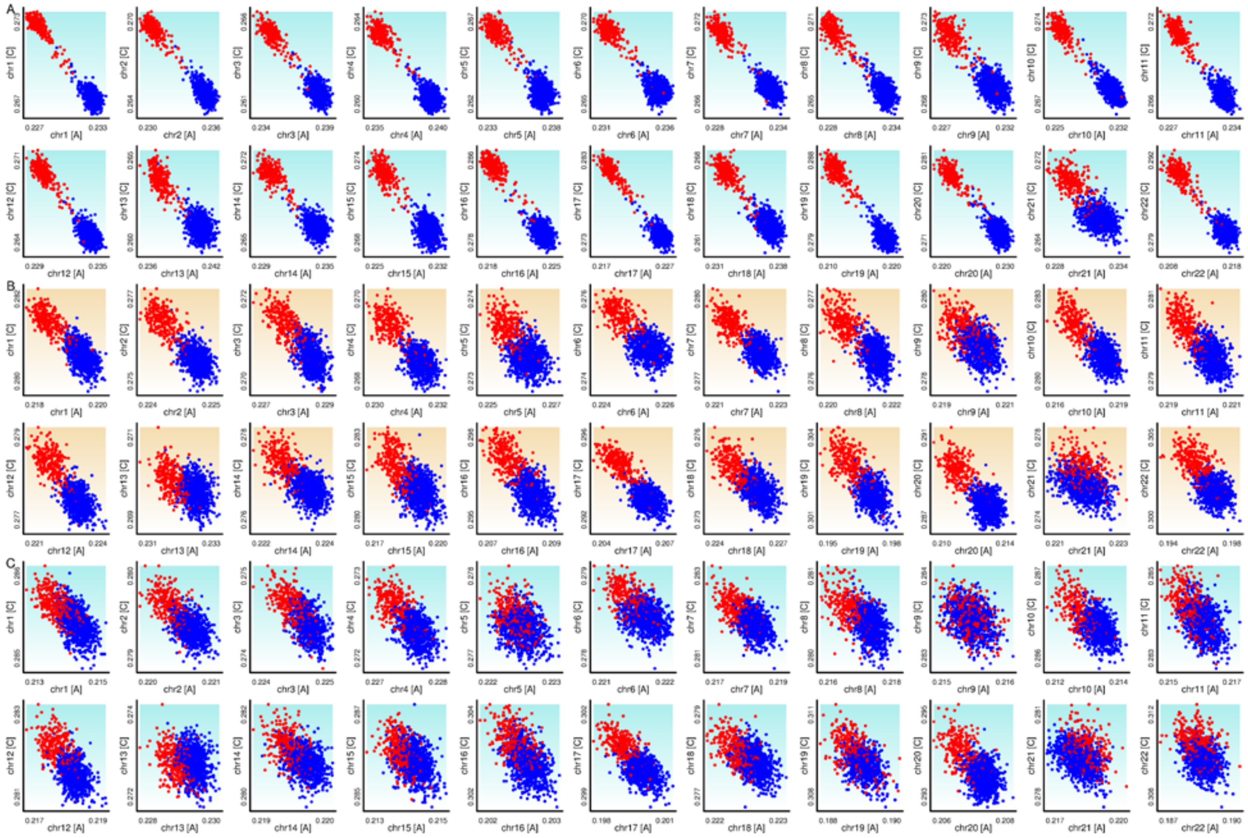

Figure S4. Base composition conformity to PR2 and AT-increase pattern at the chromosome level in humans. Chromosome-wide, individual-strand [A] and [C] are plotted for autosomal chromosomes to show PR2 ([A] ≈ [T], [G] ≈ [C], and [A] + [C] ≈ 0.5), and [A] increase from African-ancestry (red dots) to non-African-ancestry (blue dots) samples. (**A**) 7.0M SNPs with MAF >= 5%, (**B**) 11.6M SNPs with MAF >= 5%, (**C**) 15.4M SNPs with MAF > 0.5%.

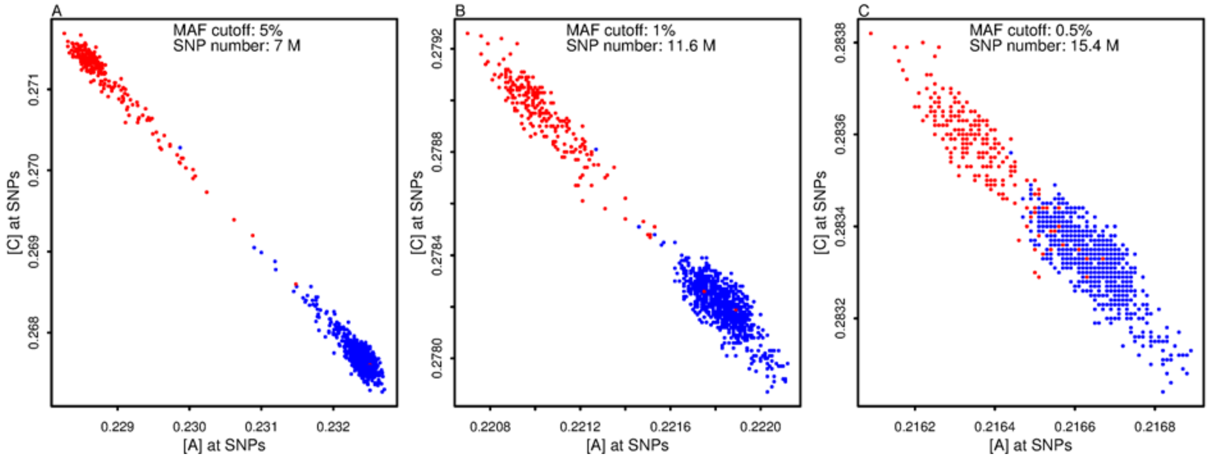

Figure S5. The PR2 and A&T-increase pattern of base composition across SNPs are consistent at different minor-allele frequency (MAF) cutoffs. Genome-wide, individual-strand [A] and [C] are plotted to show PR2 ([A] ≈ [T], [G] ≈ [C], and [A] + [C] ≈ 0.5), and [A] increase from African-ancestry (red dots) to non-African-ancestry (blue dots) samples. (**A**) 7.0M SNPs with MAF >= 5%, (**B**) 11.6M SNPs with MAF >= 5%, (**C**) 15.4M SNPs with MAF > 0.5%. Including SNPs with low-frequency and rare allele changes the base composition of African ancestry and non-African-ancestry individuals, and the difference between two populations. Within the sets of additional SNPs (4.6M with MAF 1%~5%, and 3.8M with MAF 0.5%~1%), [A] ≈ [T], [G] ≈ [C], and [A]&[T]>[G][C] are also evident. As more of these SNPs being counted in calculating [A] (from 5% to 1% to 0.5% cut-offs), individual [A] values decrease (from **panel A** to **panel B** to **panel C**) because that major alleles are more often to be G or C. Overall, both PR2 and A&T-increase patterns do not change.

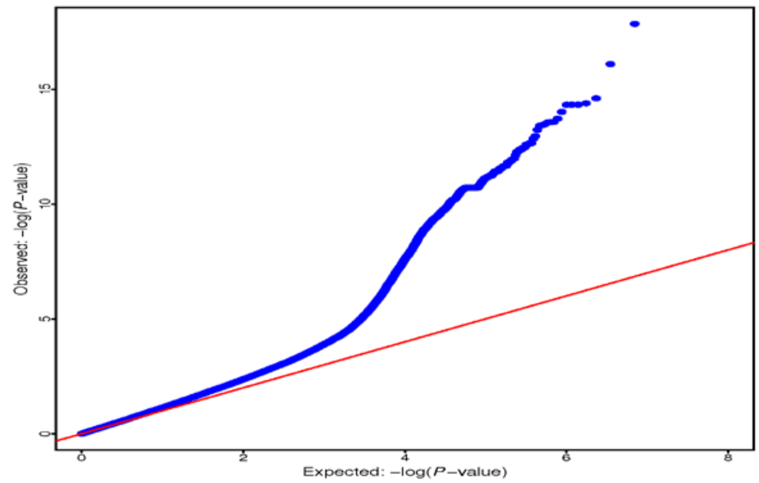

Figure S6. Quantitle-quantile plot of individual SNP tests from the genome-wide scan results with the mixed model. Bonferroni correction was applied to obtain the genome-wide threshold at $-\log_{10}$(*P*-value) = 8.1 (equivalent to *P*-value of 7×10^{-9}).

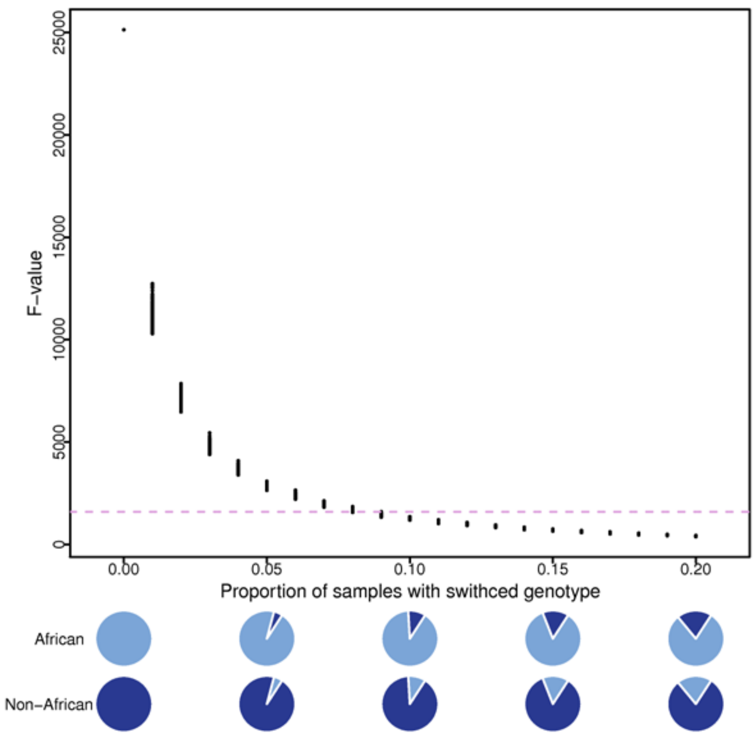

Figure S7. Simulation to determine the threshold value for interpreting the results from genome-wide scan with the linear model in the human 1000 Genomes data analysis. Different proportions of genotypes were switched from complete fixation in African-ancestry and non-African-ancestry samples to monitor the dynamic of the *F*-test statistics. A threshold *F*-value found at the elbow position of the trend curve (F=1,590) was used to distinguish the peaks of the Manhattan plot of the linear model from the background noise.

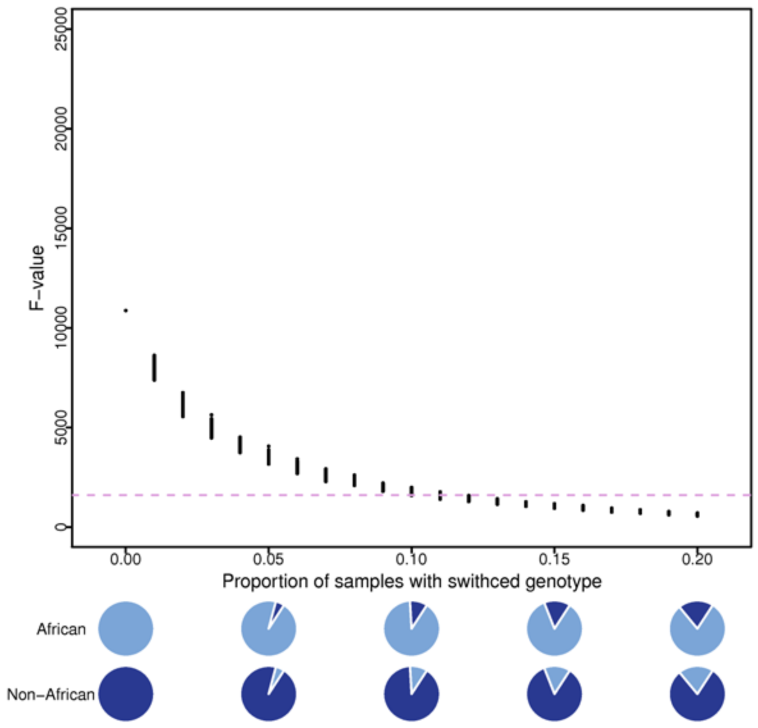

Figure S8. Simulation to determine the threshold value for interpreting the results from genome-wide scan with the linear model in the HapMap 3 data analysis. Different proportions of genotypes were switched from complete fixation in African-ancestry and non-African-ancestry samples to monitor the dynamic of the *F*-test statistics. A threshold *F*-value found at the elbow position of the trend curve (*F*=1,900) is used to distinguish the peaks of the Manhattan plot of the linear model from the background noise.

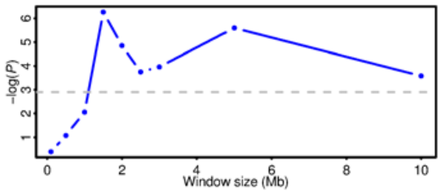

Figure S9. Enrichment tests for DNA repair genes within genomic regions identified through genome scans of base composition. Different window sizes were used to define the genomic regions. The test is to show whether genomic regions tagged by GWAS signals are more likely to contain any of the 170 known DNA repair genes, than a randomly chosen genomic region. The 1.5 Mb window size showed the most significant enrichment. The dashed line shows the threshold of enrichment test at *P*-value = 0.01.

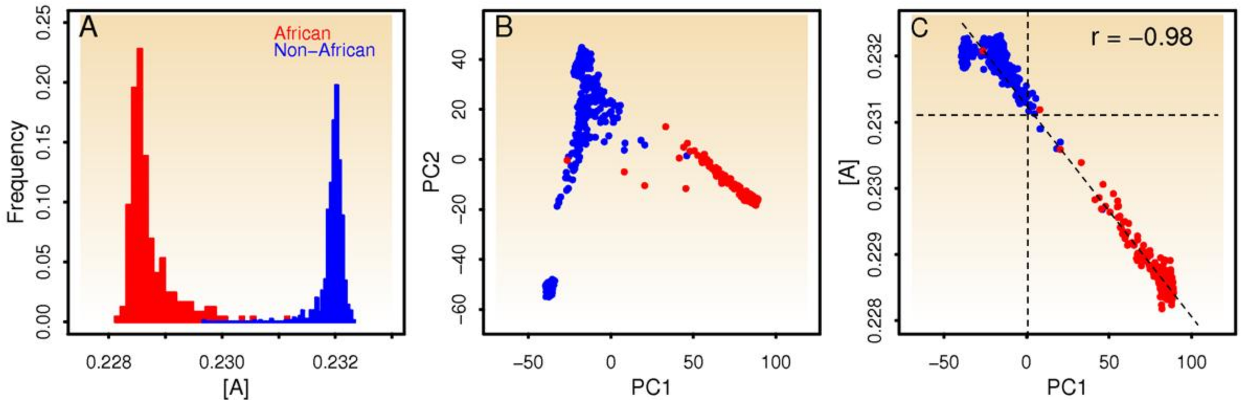

Figure S10. Base composition difference is underlying the major genome divergence in humans. (**A**) Histogram of [A] values across polymorphic sites for 1,092 humans in the 1000 Genomes data shows the overall AT-increase from African-ancestry (red) to non-African-ancestry (blue). (**B**) Principal component analysis of SNPs in the human 1000 Genomes data. Each dot represents one of 1,092 samples. The first principal component (PC1) separates African-ancestry and non-African-ancestry samples. (**C**) Base composition ([A]) across polymorphic sites correlates (*r* = -0.98) with the molecular divergence captured by PC1.

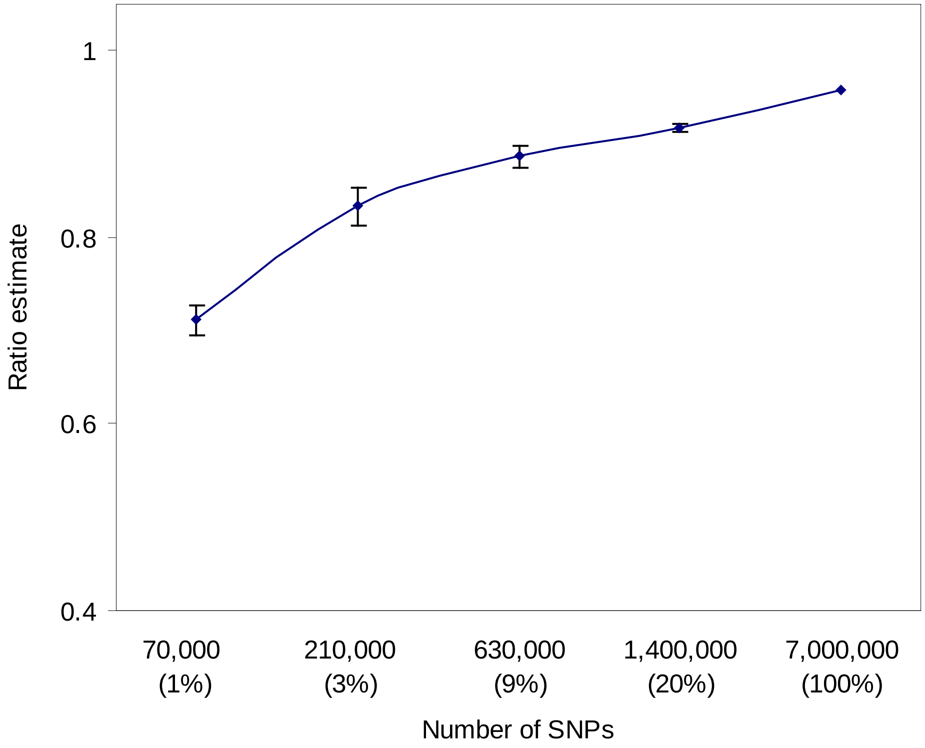

Figure S11. Ratios of additive genetic variance of base composition over the sum of additive variance and residual variance were derived with the mixed model procedure. Because [A] is a genome phenotype, we chose not to use the term heritability to avoid confusion. The overall human 1000 Genomes data were used in the analysis. From the total 7M SNPs, we randomly sampled different proportions of SNPs for the [A] calculation, and these difference sets of phenotype values were then fitted in the mixed model with a constant additive relationship matrix derived from the marker-based kinship matrix. Keeping the same kinship matrix is so that we can demonstrate the impact of the number of SNPs on the [A] values, and subsequently the variance component estimation. A set of 10 subsampling runs was conducted.

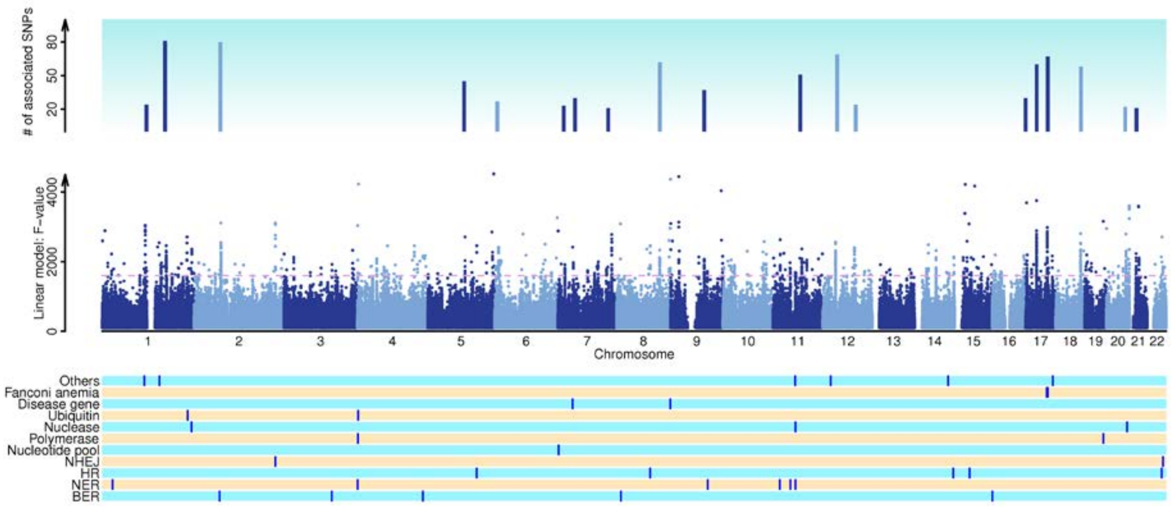

Figure S12. Second genome-wide scan with a linear model to identify genomic regions underlying variation in base composition. The upper panel displays the genomic regions (1 Mb) with 20 or more Trait Associated SNPs (TASs) detected by the linear model using genome-wide [A] as trait values from 1,092 humans. The middle panel shows the association signals detected by the linear model between the genome-wide [A] values across polymorphic sites and individual SNPs. The F-test value is plotted on the y-axis to indicate the signal strength; the *P*-value is too small to obtain because of the large sample size and test statistic values. The dashed line indicates the threshold suggested by simulation to control for false positives. The lower panel shows the position and pathway information of known genes (blue bars) from different DNA repair mechanisms flagged by TASs. NHEJ, nonhomologous end joining; HR, homologous recombination; NER, nucleotide excision repair; and BER, base excision repair. Please note that SNP rs2814778, an ancestry informative marker with the highest F value (9070), was excluded in the Manhattan plot because of no other nearby SNPs having strong signals. Analysis indicated that high association of this SNP may be caused by its overall high linkage disequilibrium value with a few other loci distributed across the genome.

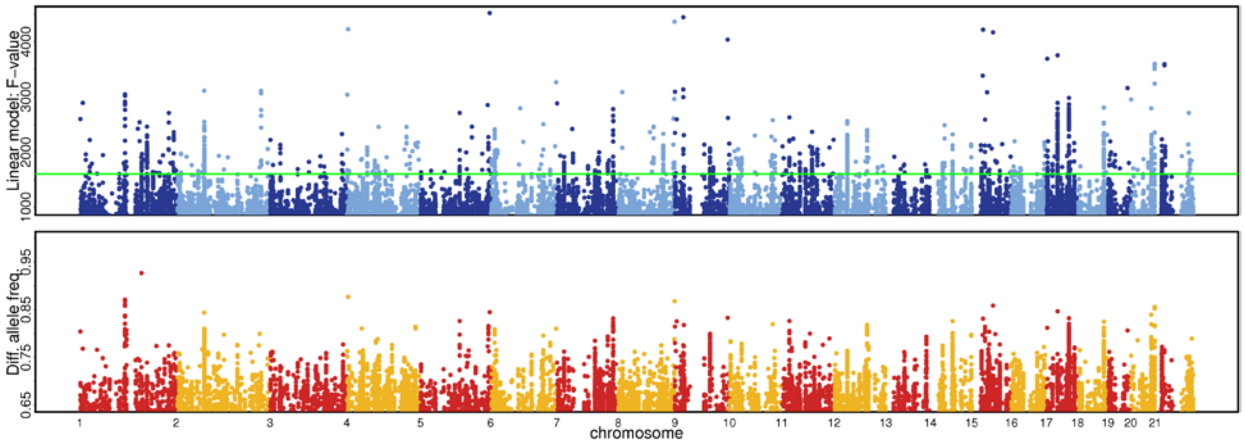

Figure S13. Consistency between allele frequency differences and results of genome scan with the linear model. Absolute values of allele frequency differences between African-ancestry and non-African-ancestry groups were calculated for the allele with a minor frequency within the African-ancestry group.

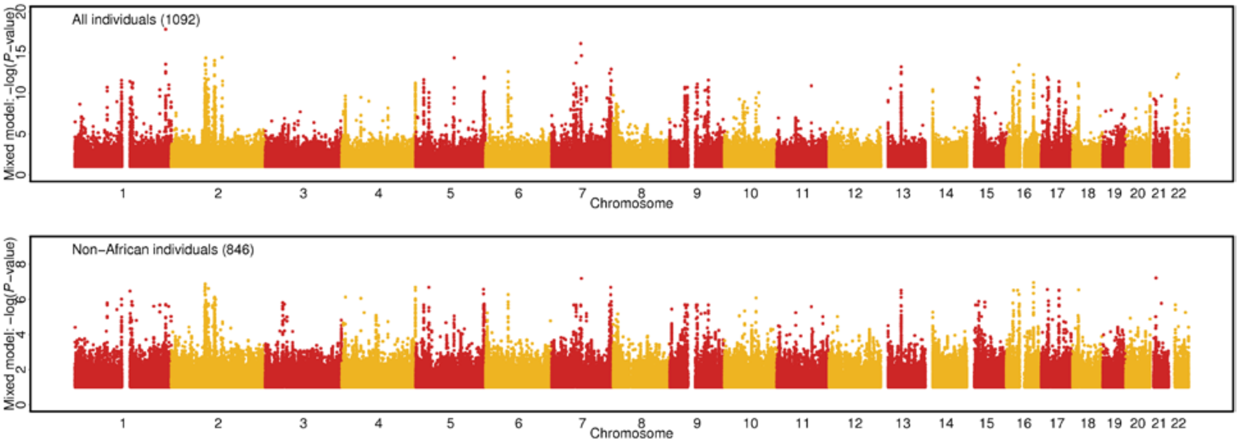

Figure S14. Consistency of results across genome-wide scans with mixed models, controlling for both population structure (fixed effect) and relatedness (random effect). Results from analysis of all human 1000 Genomes samples (n = 1,092) and only samples of non-African ancestry (n_1 = 846) are shown. The non-African-ancestry group has a larger sample size than the African-ancestry group (n_2 = 246) and so yielded more pronounced association signals.

Table S1. Whole-genome level base composition across 2,210 sequenced genomes. *(**NOTE**: This table is submitted in a separate Excel file due to its large size)*

Table S2. Data information of the eight species groups from which the conformity to PR2 across polymorphic sites was observed, as was the AT-increase pattern from the basal to derived groups. Basal and derived groups were classified according to the original publications.

Species	Samples	SNPs	Basal group	Derived group	*P*-value†	Refs
Human	1,092 (1000 Genomes)	7,003,981	ASW, LWK, YRI	CEU, CHB, CHS, CLM, FIN, GBR, IBS JPT, MXL, PUR, TSI	~ 0 ($<1\times10^{-280}$)	(1)
Dog	456	283,574	ShP, GSl, Eur	BeT, Bgl, BMD, BoC, BoT, BrS, CoS, Dac, Dob, EBD, Elk, ESt, FSp, GoS, GRe, Gry, GSh, IrW, JRT, LRe, NFd, NSD, Rtw, Sci, StP, TYo, Wei	4.5×10^{-26}	(2)
Mouse	31	421,673	*Mus musculus musculus* (Wild-caught)	*M. musculus musculus* (Laboratory)	1.2×10^{-8}	(3)
Silkworm	40	3,252,850	*Bombyx mandarina*	*B. mori*	4.7×10^{-8}	(4)
Rice	50	1,954,117	*Oryza rufipogon*, *O. nivara*	*O. sativa*	1.7×10^{-4}	(5)
Maize	46	9,230,417	*Zea mays parviglumis*	*Z. mays mays*	8.4×10^{-13}	(6)
Soybean	31	2,639,892	*Glycine soja*	*G. max*	3.5×10^{-5}	(7)
Grape	17	382,368	*Vitis sylvestris*, *V. amurensis*, *V. cinerea*, *V. labrusca*, *V. palmata*, *V. rotundifolia*	*V. vinifera*	1.2×10^{-4}	(8)

†, *P*-value from the two-sample t-test of [A], basal *versus* derived. Because of the large sample size in humans, the *P*-value is essentially 0.

Table S3. Data information from spontaneous mutation-accumulation experiments and induced-mutation experiments for analyzing PR2 and AT-increase across mutation sites.

Species	Spontaneous / mutagen	Ref
Escherichia coli	NTG	(9)
Caenorhabditis elegans	Spontaneous	(10)
	EMS; ENU; or UV/TMP induced	(11)
Drosophila melanogaster	Spontaneous	(12)
Arabidopsis thaliana	Spontaneous	(13)
	Regenerant	(14)
	Fast neutron	(15)
Oryza sativa	Regenerant	(16)
	EMS	(17)
Mus musculus	ENU	(18)

*(**NOTE**: The following three tables are submitted in different Excel files due to their large sizes)*

Table S4. Overrepresented GO terms with genomic regions tagged GWAS signals. A series of window sizes were used to define the genomic region and the enrichment tests were conducted for each GO term.

Table S5. The list of known genes from different DNA repair pathways and the trait-associated SNPs (TASs) detected by the genome-wide scans with the linear model and the mixed model. The order follows the chromosome and physical position.

Table S6. Genome-wide scan by the mixed model detected SNPs that have a -$\log_{10}$(P-value) above the threshold determined by Bonferroni correction. Genome-wide scan by the linear model detected SNPs that have an F-value above the threshold determined by simulation.

1. Abecasis GR, *et al.* (2012) An integrated map of genetic variation from 1,092 human genomes. *Nature* 491:56-65.
2. Vaysse A, *et al.* (2011) Identification of genomic regions associated with phenotypic variation between dog breeds using selection mapping. *PLoS Genetics* 7:e1002316.
3. Yang H, *et al.* (2011) Subspecific origin and haplotype diversity in the laboratory mouse. *Nature Genetics* 43:648-655.
4. Xia Q, *et al.* (2009) Complete resequencing of 40 genomes reveals domestication events and genes in silkworm (Bombyx). *Science* 326:433-436.
5. Xu X, *et al.* (2011) Resequencing 50 accessions of cultivated and wild rice yields markers for identifying agronomically important genes. *Nature Biotechnology* 30:105-111.
6. Chia JM, *et al.* (2012) Maize HapMap2 identifies extant variation from a genome in flux. *Nature Genetics* 44:803-807.
7. Lam HM, *et al.* (2010) Resequencing of 31 wild and cultivated soybean genomes identifies patterns of genetic diversity and selection. *Nature Genetics* 421053-1059.
8. Myles S, *et al.* (2010) Rapid genomic characterization of the genus *vitis*. *PloS One* 5:e8219.
9. Lee DH, Feist AM, Barrett CL, & Palsson BO (2011) Cumulative number of cell divisions as a meaningful timescale for adaptive laboratory evolution of *Escherichia coli*. *PloS One* 6:e26172.
10. Denver DR, *et al.* (2009) A genome-wide view of *Caenorhabditis elegans* base-substitution mutation processes. *Proceedings of the National Academy of Sciences of the United States of America* 106:16310-16314.
11. Flibotte S, *et al.* (2010) Whole-genome profiling of mutagenesis in *Caenorhabditis elegans*. *Genetics* 185:431-441.
12. Keightley PD, *et al.* (2009) Analysis of the genome sequences of three *Drosophila melanogaster* spontaneous mutation accumulation lines. *Genome Research* 19:1195-1201.
13. Ossowski S, *et al.* (2010) The rate and molecular spectrum of spontaneous mutations in *Arabidopsis thaliana*. *Science* 327:92-94.
14. Jiang C, *et al.* (2011) Regenerant *Arabidopsis* lineages display a distinct genome-wide spectrum of mutations conferring variant phenotypes. *Current Biology* 21:1385-1390.

15. Belfield EJ, *et al.* (2012) Genome-wide analysis of mutations in mutant lineages selected following fast-neutron irradiation mutagenesis of *Arabidopsis thaliana*. *Genome Research* 22:1306-1315.
16. Miyao A, *et al.* (2012) Molecular spectrum of somaclonal variation in regenerated rice revealed by whole-genome sequencing. *Plant & Cell Physiology* 53:256-264.
17. Abe A, *et al.* (2012) Genome sequencing reveals agronomically important loci in rice using MutMap. *Nature Biotechnology* 30:174-178.
18. Beutler B (2012) MUTAGENETIX, http://mutagenetix.utsouthwestern.edu/.
